# Dynamics of the infant gut microbiota in the first 18 months of life: the impact of maternal HIV infection and breastfeeding

**DOI:** 10.1186/s40168-022-01230-1

**Published:** 2022-04-12

**Authors:** Silvia Grant-Beurmann, Jibreel Jumare, Nicaise Ndembi, Olayemi Matthew, Ashley Shutt, Augustine Omoigberale, Olivia A. Martin, Claire M. Fraser, Man Charurat

**Affiliations:** 1 Institute for Genome Sciences, University of Maryland School of Medicine, Baltimore, MD USA; 2 Institute of Human Virology, University of Maryland School of Medicine, Baltimore, MD USA; 3 Institute of Human Virology, Abuja, Nigeria; 4 University of Benin Teaching Hospital, Edo, Nigeria; 5 Department of Surgery, University of Maryland School of Medicine, Baltimore, MD USA; 6 Department of Medicine, University of Maryland School of Medicine, Baltimore, MD USA

**Keywords:** HIV-exposed infants, Gut microbiota, Breast milk metabolome, Antiretroviral therapy, Breastfeeding, *Bifidobacterium*, Adverse growth outcome, Weight-for-age *z*-score, Acylcarnitine, Kynurenine

## Abstract

**Background:**

Access to antiretroviral therapy (ART) during pregnancy and breastfeeding for mothers with HIV has resulted in fewer children acquiring HIV peri- and postnatally, resulting in an increase in the number of children who are exposed to the virus but are not infected (HEU). HEU infants have an increased likelihood of childhood infections and adverse growth outcomes, as well as increased mortality compared to their HIV-unexposed (HUU) peers. We explored potential differences in the gut microbiota in a cohort of 272 Nigerian infants born to HIV-positive and negative mothers in this study during the first 18 months of life.

**Results:**

The taxonomic composition of the maternal vaginal and gut microbiota showed no significant differences based on HIV status, and the composition of the infant gut microbiota at birth was similar between HUU and HEU. Longitudinal taxonomic composition of the infant gut microbiota and weight-for-age *z*-scores (WAZ) differed depending on access to breast milk. HEU infants displayed overall lower WAZ than HUU infants at all time points. We observed a significantly lower relative abundance of *Bifidobacterium* in HEU infants at 6 months postpartum. Breast milk composition also differed by time point and HIV infection status. The antiretroviral therapy drugs, lamivudine and nevirapine, as well as kynurenine, were significantly more abundant in the breast milk of mothers with HIV. Levels of tiglyl carnitine (C5) were significantly lower in the breast milk of mothers without HIV. ART drugs in the breast milk of mothers with HIV were associated with a lower relative abundance of *Bifidobacterium longum.*

**Conclusions:**

Maternal HIV infection was associated with adverse growth outcomes of HEU infants in this study, and these differences persist from birth through at least 18 months, which is a critical window for the development of the immune and central nervous systems. We observed that the relative abundance of *Bifidobacterium* spp. was significantly lower in the gut microbiota of all HEU infants over the first 6 months postpartum, even if HEU infants were receiving breast milk. Breastfeeding was of benefit in our HEU infant cohort in the first weeks postpartum; however, ART drug metabolites in breast milk were associated with a lower abundance of *Bifidobacterium*.

**Video abstract**

**Supplementary Information:**

The online version contains supplementary material available at 10.1186/s40168-022-01230-1.

## Background

Improved access to antiretroviral therapy (ART) for mothers with HIV during pregnancy and breastfeeding has resulted in fewer children acquiring HIV peri- and postnatally [1]. There has been a resultant increase in the number of children born to mothers with HIV who are exposed to the virus, but who are not infected (HEU). This population is estimated to be 14.8 million children globally; with approximately 13.2 million children living in sub-Saharan Africa [2]. It has been shown previously that HEU infants have an increased likelihood of childhood infections and adverse growth outcomes, as well as increased mortality compared to their HIV unexposed (HUU) peers [3–7].

Exposure to HIV *in utero* has been shown to impact the gut microbiota of HEU infants [8–11], pointing to a potential link between maternal HIV status, the infant gut microbiota, and infant health. Perturbations in the infant gut microbiota have been linked with altered immunity, and increased susceptibility to disease [12–16]. In addition, the breast milk microbiota has been previously found to differ between mothers with and without HIV [10] and survival of HEU infants in Africa has been associated with breast milk oligosaccharide composition [17]. These breast milk oligosaccharides, in turn, have been linked to the gut microbiota of HEU [8]. Taken together, these findings provide considerable evidence that maternal HIV status has a profound impact on the acquisition and subsequent development of the infant gut microbiota [18–21].

We set out to further investigate the relationship between in utero HIV exposure and adverse growth outcomes in HEU by conducting a longitudinal study of the maternal and infant microbiota of 272 Nigerian mother-infant pairs, as well as the breast milk metabolome. We hypothesized that acquisition of an altered gut microbiota from a mother with HIV, further exacerbated by differences in breast milk composition between mothers with and without HIV, negatively impacts growth and increases the risk of adverse clinical outcomes among HEU infants.

## Results

### Most characteristics of mother-infant pairs were similar regardless of motherʼs HIV status

Table 1 shows the baseline characteristics of the HEU and HUU infants and their mothers. The median age of mothers was 32 years, similar for women with and without HIV (*P* = 0.12). Most of the mothers were employed (87.1%), with no significant difference between the two groups (*P* = 0.853). The mothers of the HEU children were more likely to have lower levels of education (*P* < 0.001), less likely to be married (*P* < 0.001), but more likely to be multiparous (*P* = 0.039). While there was a slightly greater proportion of deliveries via cesarean section (33.8% vs. 28.5%) among the women with HIV, this did not reach statistical significance (*P* = 0.418). The women with HIV had a median CD4 count of 429 cells/ml (IQR: 285–566) at enrollment. The median birth weight was significantly lower among HEU babies (*P* < 0.001), but the proportion of those born with low birth weight (< 2.5 Kg) and those born prematurely did not differ significantly between HEU and HUU children. Weight-for-age *z*-scores (WAZ) were significantly lower among HEU as compared to HUU babies at birth. The median duration of breastfeeding was 9 months, and 46% of infants were exclusively breastfed for 6 months; however, in HEU infants there was a significantly shorter duration of breastfeeding (*P* < 0.001) as well as lower proportion of exclusively breastfed children (*P* < 0.001). As expected, and consistent with standard recommendations, the use of TMP-SMX was significantly greater among HEU infants at all follow-up timepoints (*P* < 0.001). This pattern was similar for overall antibiotic prescription until the 9-month visit, beyond which the difference between HEU and HUU was not statistically significant. The variable “antibiotic use” captured any antibiotic prescribed to the participants during their clinic visit. It reflected trimethoprim-sulfamethoxazole (TMP-SMX), other antibiotics, or a combination of these, thereby enabling us to account for any antibiotic use in the analyses. During the 18-month study period, none of the infants included in this analysis became HIV-positive.

**Table 1:** Baseline maternal and infant characteristics

	***N***	All	HIV-/HUU	HIV+/HEU	
			131	141	***P*** value
**Maternal**					
** Maternal Age (years), median (IQR)**	263	32 (29, 36)	32 (29, 35)	32 (29, 37)	0.1248^W^
** Employed,*n*(%)**	263	229 (87.1)	110 (86.6)	119 (87.5)	0.8527^F^
** Education,*n*(%)**	263				< .0001^F^
** Primary/junior secondary**		47 (17.9)	3 (2.4)	44 (32.4)	
** Senior secondary**		81 (30.8)	26 (20.5)	55 (40.4)	
** Tertiary**		135 (51.3)	98 (77.2)	37 (27.2)	
** Married,*n*(%)**	263	213 (81.0)	124 (97.6)	89 (65.4)	< .0001^F^
** Parity,*n*(%)**	262				0.0388^F^
** Primip**		74 (28.2)	44 (34.9)	30 (22.1)	
** 2–4**		181 (69.1)	80 (63.5)	101 (74.3)	
** > 4**		7 (2.7)	2 (1.6)	5 (3.7)	
** CD4 Count (cells/mm**^**3**^**), median (IQR)**	136			428.5 (285, 566)	
**Baby**					
** Gender, female,*n*(%)**	272	118 (43.5)	60 (45.8)	58 (41.4)	0.4496^F^
** Delivery type,*n*(%)**	272				0.4183^F^
** Vaginal**		186 (68.4)	92 (70.2)	94 (66.7)	
** Cesarean section**		86 (31.6)	39 (29.8)	47 (33.3)	
** Premature delivery,*n*(%)**	268	20 (7.5)	7 (5.4)	13 (9.4)	0.2486^F^
** Birth weight (Kg), median (IQR)**	271	3 (2.7, 3.4)	3.2 (2.8, 3.5)	2.9 (2.5, 3.25)	< .0001^W^
** Birth weight <2.5 Kg,*n*(%)**	271	33 (12.2)	12 (9.2)	21 (15.0)	0.1793^F^
**Anthropometrics at birth,*z*-score, mean (SD)**					
** Weight-for-age**	271	− 0.71 (1.2)	0.39 (1.2)	− 1.01 (1.2)	< .0001^T^
** Meconium microbiome Shannon diversity, median (IQR)**	176	1.75 (1.2, 2.3)	1.71 (1.2, 2.2)	1.78 (1.1, 2.3)	0.7386^W^
** Exclusive breastfeeding (6 months),*n*(%)**	246	112 (45.5)	72 (62.1)	40 (30.8)	< .0001^F^
** Breastfeeding duration (months), median (IQR)**	272	6 (1, 9)	9 (9, 15)	1 (1, 6)	< .0001^W^
** Trimethoprim-sulfamethoxazole use (6 months),*n*(%)**	246	131 (53.3)	15 (12.9)	116 (89.2)	< .0001^W^
** Antibiotic use (6 months),*n*(%)**	257	111 (43.2)	35 (29.7)	76 (54.7)	< .0001^W^

^W^Wilcoxon’s, ^F^Fisher’s, ^T^*t* test

*HUU*, HIV-unexposed uninfected; *HEU*, HIV-exposed uninfected; *IQR*, interquartile range; *N*, number of participants; *SD*, standard deviation

### Maternal vaginal and infant gut microbiomes increased in diversity over time

High-throughput sequencing of the hypervariable regions V3 and V4 of the 16S ribosomal RNA gene was used to characterize the taxonomic composition of the samples collected in this study. Two sample types from the mothers (vaginal swabs; MVS and stool; MST) were collected prior to (at time of enrollment, after 12 weeks of gestation) and at birth (number of MST samples collected at birth was lower in comparison to prenatally due to fewer specimens being produced at that time point). Infant meconium (IMC) was collected at birth, and infant stool samples (IST) were collected at 6 weeks, 6 months, 9 months, 15 months, and 18 months postpartum (Table S1).

**Fig. 1:**
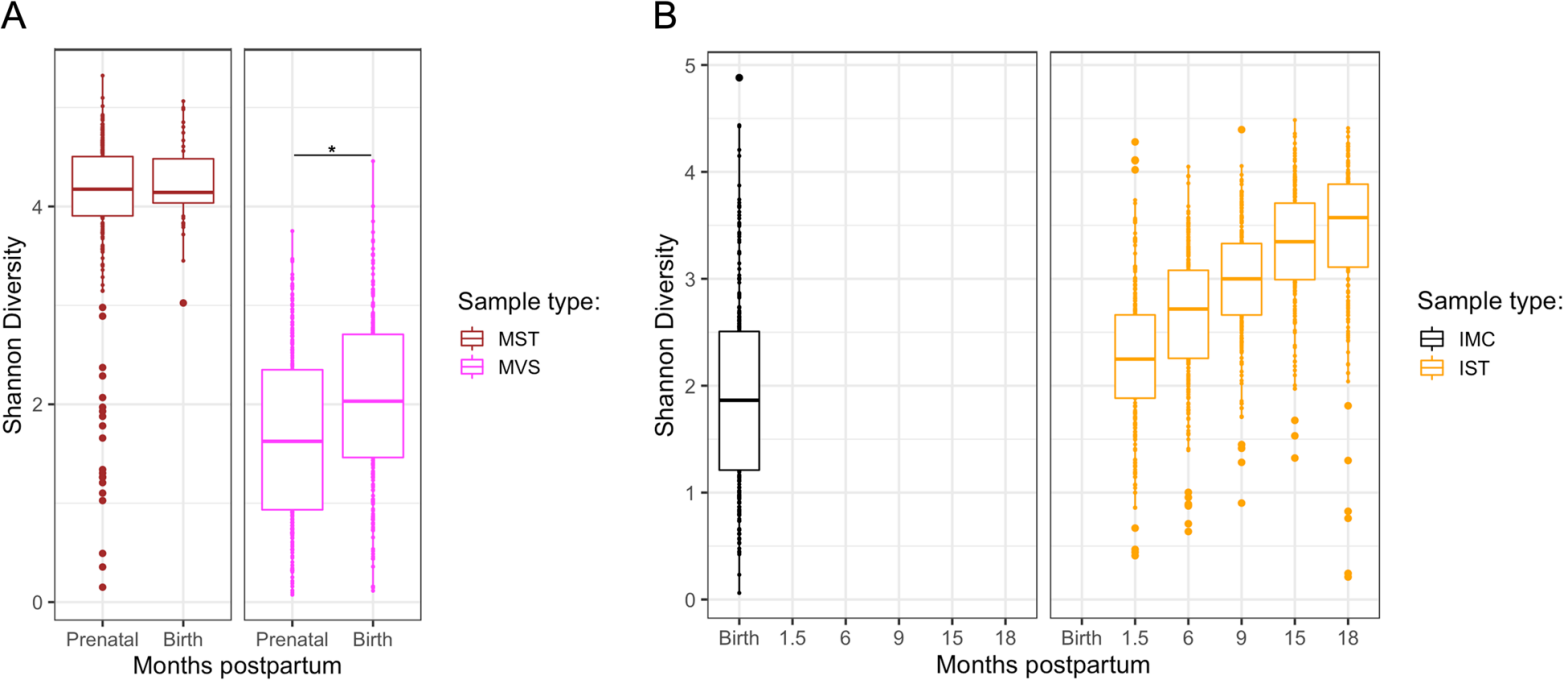
Alpha diversity (Shannon index) for each sample type and timepoint. **A** Maternal stool (MST) microbiome diversity did not change prenatally to birth (Shannon index, *P* = 0.138), whereas maternal vaginal (MSV) microbiome diversity significantly increased after birth (Shannon index, *P* < 0.001). **B** Infant meconium (IMC) and infant stool (IST) microbiome diversity displayed significant increases from birth to 18 months postpartum (Shannon index, *P* < 0.001). Horizontal lines in boxplots indicate median; boxes show first and third quartiles

We first looked broadly at the average microbiome composition at each sample site over all time points. Principal coordinates analysis (PCoA) based on Bray-Curtis dissimilarity revealed a distinct clustering of the mothers’ vaginal and stool samples (PERMANOVA, *P* = 0.001; Fig. S1A). Consistent with this observation was the finding that the alpha diversity between these samples was significantly different with MST exhibiting a higher diversity (Shannon index; SI = 4.05 ± 0.05 SEM) in comparison to MVS (SI = 1.85± 0.04 SEM) (Fig. S2A; Shannon index, *P* < 0.001). Vaginal microbiome diversity significantly increased after birth (Fig. 1A; Shannon index, *P* < 0.001), while maternal stool microbiome diversity was unchanged over this same time period (Fig. 1A; Shannon index, *P* = 0.138).

The two sample types for the infants also displayed significant differences (Fig. S1B). In the aggregate, IMC had a lower diversity, (SI = 1.92 ± 0.05 SEM) compared to IST (SI = 2.89 ± 0.02 SEM) (Fig. S2B; Shannon index, *P* < 0.001). As previously reported, we observed a significant increase in the diversity of IST from birth to 18 months postpartum (Fig. 1B; Shannon index, *P* < 0.001).

### Maternal microbiome composition showed no significant differences based on HIV status

To determine the impact of HIV infection on the maternal microbiota, the taxonomic composition of the microbiota from mothers with HIV was compared to that from mothers without HIV. PcoA revealed no clear separation between mothers with and without HIV for either of the sample sites (PERMANOVA, *P* = 0.876; Fig. S3). There were also no significant differences in bacterial diversity identified based on HIV infection (Fig. S4; Shannon index, *P* = 0.672).

Longitudinal changes in the microbiota of mothers with and without HIV were also investigated. No significant differences in the stool or vaginal microbiota between mothers with and without HIV (Fig. S5A) were found. The maternal stool microbiota contained both *Bacteroides* and *Prevotella* from the phylum Bacteroidetes, several genera in the Firmicutes phylum including *Blautia*, *Faecalibacterium*, *Lactobacillus*, *Roseburia*, *Staphylococcus* and *Streptococcus*, and *Bifidobacterium*. The maternal vaginal microbiota was dominated by *Lactobacillus*, along with *Gardnerella* and *Pseudomonas*; however, in mothers with HIV, the abundance of *Lactobacillus* was lower (prenatal; 63% vs. 72%, birth; 51% vs. 55%) and the abundance of *Gardnerella* was higher (prenatal; 17% vs. 11%, birth; 13% vs. 12%) compared to mothers without HIV (Fig. S5B), however, these differences did not reach statistical significance. At none of the timepoints was the bacterial diversity significantly different (Shannon index, *P* > 0.05) between mothers with and without HIV for any of the sample sites (Shannon index, MVS—prenatal; *P* = 0.076, MVS—birth; *P* = 0.461, MST—prenatal; *P* = 0.761, MST – birth; *P* = 0.312, Fig. S5C).

### Breastfeeding status is associated with differences in the gut microbiota of infants

At the time of this study, the World Health Organization (WHO) recommendation for mothers with HIV was to exclusively breastfeed for 6 months; introduce complementary feeds afterwards, while continuing to breastfeed for up to 24 months [22]. This recommendation was most relevant in situations where ART was available to guarantee the best chance for HIV-free survival for exposed infants in resource-limited settings. However, many of the mothers with HIV in this study opted not to breastfeed for long durations due to concerns for transmission of HIV to their babies (Table 1, Fig. S6). At 6 and 9 months postpartum, the majority (99% and 95%, respectively) of HUU infants were still being breastfed, whereas only 39% and 17% of HEU infants were receiving breast milk at these timepoints. As a result, the cohort was stratified not just by HIV exposure status, but also by breastfeeding status.

**Fig. 2:**
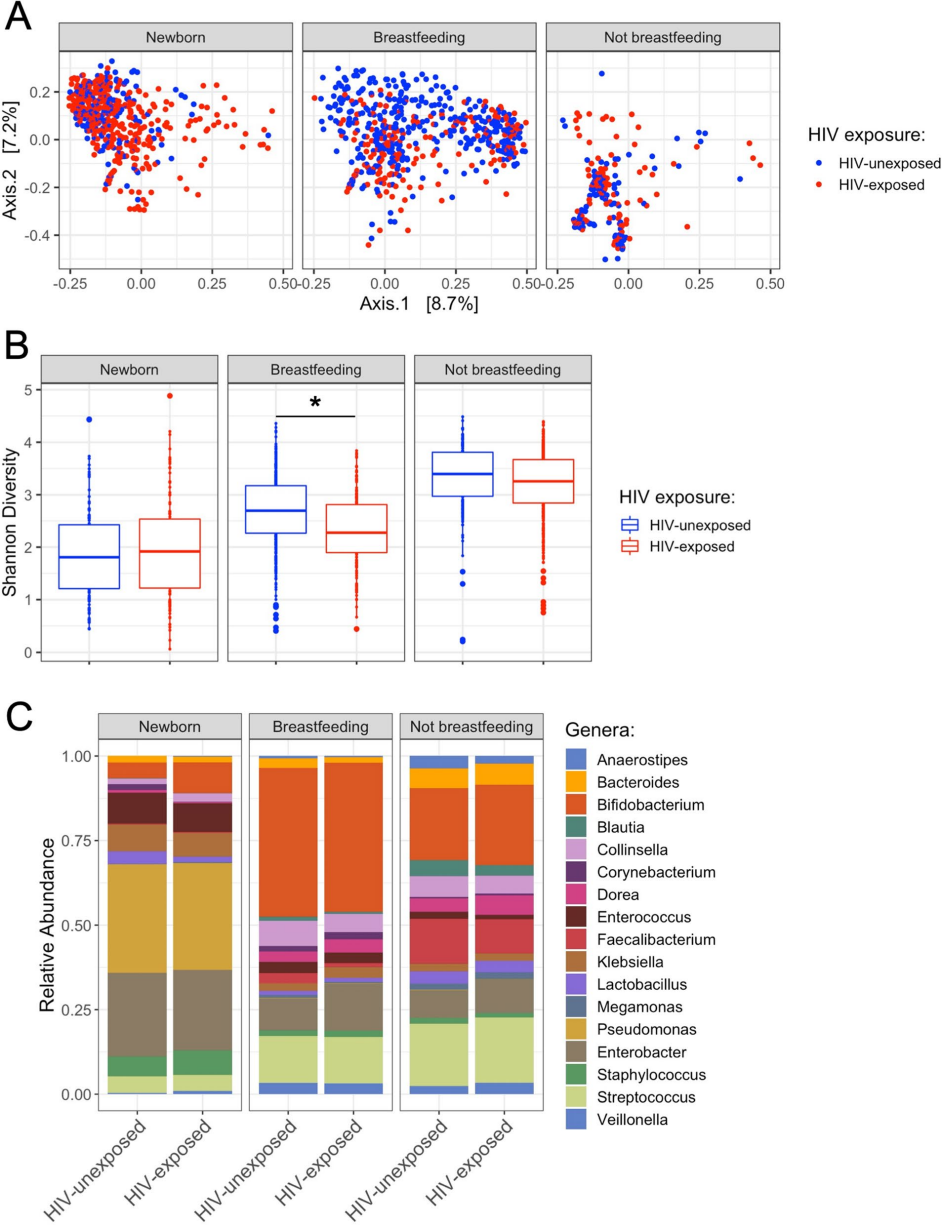
The gut microbiota in breastfeeding infants from mothers with HIV differs from that seen in mothers without HIV. For these analyses, data across all time points were aggregated based on maternal HIV and/or breastfeeding status. **A** PCoA comparing HEU to HUU infants exhibited no significant separation within breastfeeding groups (PERMANOVA, *P* = 0.459). **B** The bacterial diversity of breastfeeding HEU infants (SI = 2.33 ± 0.05 SEM) and breastfeeding HUU infants (SI = 2.67 ± 0.04 SEM) showed a significant difference (Shannon index, *P* < 0.001). There were no significant differences in the Shannon diversity between HEU (SI = 1.97 ± 0.07 SEM) and HUU (SI = 1.88 ± 0.07 SEM) infants at birth (newborn); similarly, there was no differences between HEU infants (SI = 3.19 ± 0.04 SEM) and HUU infants (SI = 3.32 ± 0.06 SEM) infants that are not breastfeeding. **C** None of the bacterial taxa relative abundances differences reached significance between the microbiota of HEU and HUU infants (only genera made up with ASVs with a mean greater than 0.5% are shown)

In newborn infants that had not yet commenced breastfeeding, meconium samples were dominated by *Pseudomonas*, *Enterobacter*, *Klebsiella*, and *Corynebacterium*. By contrast, the taxonomic composition of all infant stool samples collected at various time points postpartum was characterized by a high relative abundance of *Bifidobacterium*, *Streptococcus*, and *Enterobacter*. The relative abundances of several bacterial taxa significantly differed based on breastfeeding status as determined by MaAsLin2; *Bifidobacterium* (FDR; *P* < 0.001) and *Collinsella* (FDR; *P* = 0.040) were positively associated with breastfeeding [23], whereas *Faecalibacterium* (FDR; *P* = 0.007) and *Streptococcus* (FDR; *P* = 0.803) were negatively associated with breastfeeding. PcoA of stool samples obtained from HEU vs. HUU infants exhibited no clear separation in any of the three breastfeeding groups (PERMANOVA, *P* = 0.459; Fig. 2A); however, the Shannon diversity between breastfeeding HEU (SI = 2.33 ± 0.05 SEM) and breastfeeding HUU (SI = 2.67 ± 0.04 SEM) was significantly different (Fig. 2B; Shannon index, *P* < 0.001). There were no significant differences between HEU (SI = 1.97 ± 0.07 SEM) and HUU (SI = 1.88 ± 0.07 SEM) infants’ alpha diversity at birth (not yet breastfeeding); similarly, there was no difference between HEU (SI = 3.19 ± 0.04 SEM) and HUU (SI = 3.32 ± 0.06 SEM) alpha diversity in infants that are not breastfeeding. None of the differences in relative abundance of bacterial taxa reached significance between the microbiota of HEU and HUU infants within any of the groups based on the breastfeeding status (Fig. 2C).

### Breastfeeding infants from mothers with HIV exhibit significantly less Bifidobacteria at 6 months postpartum

**Fig. 3:**
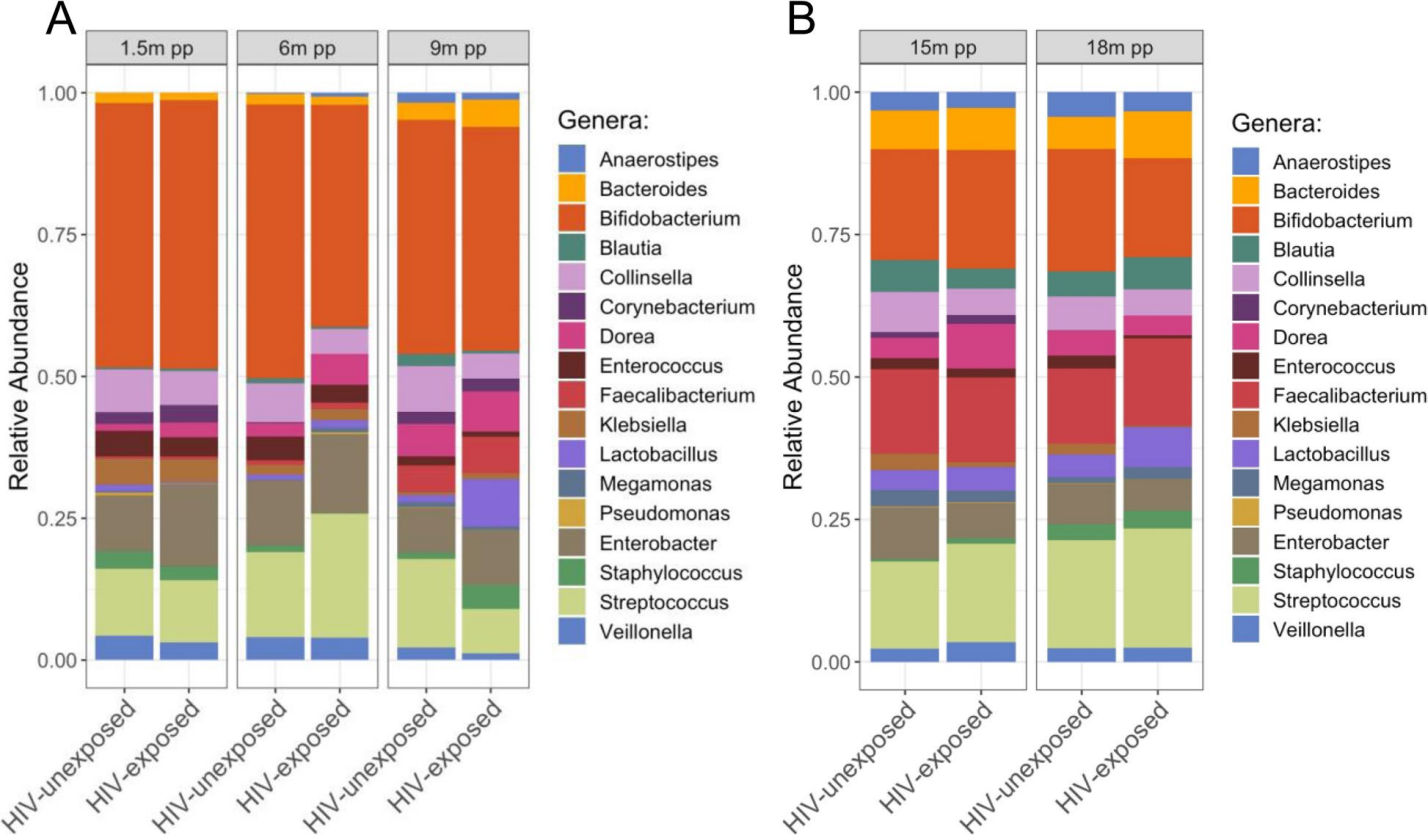
Breastfeeding infants from mothers with HIV exhibit significantly less Bifidobacteria at 6 months postpartum. **A** Bacterial taxa relative abundance differences between HEU and HUU infants within the breastfeeding cohort: *Bifidobacterium* was the only taxon that significantly differed between HEU and HUU infants at 6 months postpartum (6m pp, FDR; *P* = 0.015). **B** Bacterial taxa relative abundance differences between HEU and HUU infants within the non-breastfeeding cohort: None of the bacterial taxa relative abundance differences in the non-breastfeeding cohort reached significance between the microbiota of HEU and HUU infants. Because the majority of infants born to mothers without HIV were breastfed for 9 months, it was not possible to compare across non-breastfeeding cohorts at these earlier time points. Only genera representing ASVs with a mean greater than 0.5% are shown

Previous research has shown the importance of breastfeeding and its ability to shape the gut microbiota in early life, both directly by exposure of the neonate to the milk microbiota and indirectly, via maternal milk factors that affect bacterial growth and metabolism [24–26]. Because our data demonstrated that breastfeeding is associated with significant differences in the diversity and composition of the gut microbiota in both HEU and HUU infants [8, 10], we performed a cross-sectional comparison of the infant gut microbiota by time point. PCoA did not reveal any significantly distinct clusters based on breastfeeding status or time postpartum, although the distribution of data points was more dispersed in the breastfeeding cohort (PERMANOVA, all *P* > 0.05; Fig. S7). This heterogeneity in the distribution of data points correlated with differences in the relative abundance of *Bifidobacterium longum* (Fig. S8). Gut microbiota samples with low *B. longum* clustered to the left of the PCoA, and gut microbiota samples with high *B. longum* content were on the right. There were no significant differences in the Shannon diversity between HEU and HUU infant gut microbiota at any of the time points when grouped by breastfeeding status (Fig. S9). We did, however observe that one bacterial taxon, *Bifidobacterium*, was significantly more abundant in the breastfeeding HUU infants when compared to breastfeeding HEU infants at 6 months postpartum (Fig. 3A; FDR; *P* = 0.015). This difference was no longer observed in samples collected at 9, 15, and 18 months postpartum, which may, in part, reflect the introduction of solid foods at around 6 months of age. None of the taxa within the non-breastfeeding cohort reached statistical significance between HEU and HUU infants (Fig. 3B).

### Breastfeeding HEU infants exhibit significantly higher weight-for-age z-scores compared to non-breastfeeding HEU infants at 6 weeks postpartum

**Fig. 4:**
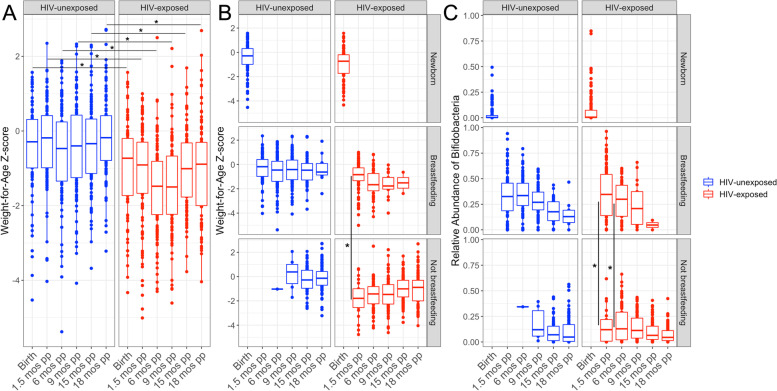
Breastfeeding HEU infants exhibit significantly higher weight-for-age *z*-scores compared to non-breastfed HEU infants at 6 weeks postpartum. **A** HEU infants have significantly lower weight-for-age *z*-scores (WAZ) compared to HUU infants at all time points (FDR; *P* < 0.001). **B** At 6 weeks postpartum, non-breastfeeding HEU infants exhibit significantly lower WAZ (− 1.82 ± 0.20) in comparison to breastfed HEU infants (− 0.99 ± 0.11) (FDR; *P* < 0.001). **C** Not breastfeeding HEU infants exhibited significantly lower relative abundances of *Bifidobacteria* at 6 weeks (15.80% ± 3.45% SEM) and 6 months (18.1% ± 1.90% SEM) postpartum when compared to breastfeeding HEU infants at 6 weeks (34.93% ± 1.53% SEM) and 6 months (28.12% ± 2.29% SEM) (*P* < 0.001 and *P* = 0.008, respectively)

Previous studies have shown that HEU infants have lower WAZ compared to HUU infants [27–30]. Our results are also consistent with those findings (Fig. 4A), which prompted us to further investigate whether there is a potential association between breastfeeding status and WAZ in HUU and HEU infants. We ran linear regression models, followed by a pairwise post hoc test, which revealed that at 6 weeks postpartum, non-breastfeeding HEU exhibit significantly lower WAZ (− 1.82 ± 0.20 SEM) in comparison to breastfed HEU infants (− 0.99 ± 0.11 SEM) (Fig. 4B, FDR; *P* < 0.001). That observation was no longer significant at 6 months postpartum or any later time points. The comparison of breastfeeding and non-breastfeeding HUU infants did not reveal any significant differences at any time point. Comparing HUU with HEU by breastfeeding status and time point demonstrated that HEU infants present with overall lower WAZ than HUU infants; newborn, breastfeeding, and non-breastfeeding HEU infants exhibit significantly lower WAZ at all time points (Fig. 4A, FDR; all *P* < 0.001).

The gastrointestinal tract of full-term healthy infants is typically dominated by the genus *Bifidobacterium* [31–33]. Then, in the first months postpartum, the loss of *Bifidobacterium* species and/or gain of other bacteria can significantly alter the maturation of the microbial community, which may lead to a variety of negative consequences for host health including a predisposition to autoimmune and metabolic diseases [34, 35]. Our data revealed a significantly lower relative abundance of *Bifidobacterium* in HEU infants at 6 months postpartum. Since breastfeeding is known to promote *Bifidobacterium* growth in the infant gut [33, 36], we explored whether the lack of breastfeeding in HEU infants during the first weeks and months postpartum was associated with a lower abundance of *Bifidobacteria*. Indeed, we did observe significantly lower relative abundances of Bifidobacteria in non-breastfed HEU infants at 6 weeks (15.80% ± 3.45% SEM) and 6 months (18.1% ± 1.90% SEM) postpartum when compared to breastfeeding HEU infants at six weeks (34.93% ± 1.53% SEM) and 6 months (28.12% ± 2.29% SEM) (*P* < 0.001 and *P* = 0.008, respectively; Fig. 4C).

To determine whether a low relative abundance of *Bifidobacteria* in HEU infants is a predictor of low WAZ, we ran a logistic regression model with breastfeeding included as a covariate. The model confirmed a link between low *Bifidobacterium* abundance and low WAZ in HEU infants (*β* = 0.09, *P* = 0.018).

### Breast milk composition differed by time point and HIV infection status

Breast milk metabolites from a subset of exclusively breastfeeding mothers with and without HIV were examined in more detail. Untargeted metabolite profiling was carried out using ultra-high-performance liquid chromatography/mass spectrometry/mass spectrometry (UHPLC/MS/MS) to characterize a wide range of metabolites (a total of 553 compounds of known identity) present in breast milk samples from 34 mothers (Table 2; 17 exclusively breastfeeding mothers with HIV and 17 exclusively breastfeeding mothers without HIV) at 6 weeks and 6 months postpartum. Table 2 shows that the 34 mothers were a good representation of the entire cohort, with the exception of parity that was not significantly different between the two groups of mothers in this subset, whereas it was significantly different in the total cohort.

**Table 2:** Baseline characteristics for the mothers and their infants selected for breast milk metabolomics

	***N***	All	HIV-/HUU	HIV+/HEU	***P*** value
			17	17	
**Maternal**					
** Maternal Age (years), median (IQR)**	34	32 (28, 35)	31 (26, 33)	32 (31, 37)	0.1239^W^
** Employed,*n*(%)**	34	30 (88.2)	15 (88.2)	15 (88.2)	1^F^
** Education,*n*(%)**	34				0.0005^F^
**Primary/junior secondary**		9 (26.5)	1 (5.9)	8 (47.1)	
**Senior secondary**		10 (29.4)	3 (17.6)	7 (41.2)	
**Tertiary**		15 (44.1)	13 (76.5)	2 (11.7)	
** Married,*n*(%)**	34	22 (64.7)	15 (88.2)	7 (41.2)	0.0104^F^
** Parity,*n*(%)**	34				0.4132^F^
**Primip**		8 (23.5)	6 (35.3)	2 (11.8)	
**2–4**		23 (67.7)	10 (58.8)	13 (76.4)	
**> 4**		3 (8.8)	1 (5.9)	2 (11.8)	
** CD4 count (cells/mm**^**3**^**), median (IQR)**	17			377 (242, 497)	
** Baby**					
** Gender, female,*n*(%)**	34	13 (38.2)	7 (41.2)	6 (35.3)	1^F^
** Delivery type,*n*(%)**	34				1^F^
**Vaginal**		28 (82.4)	28 (82.4)	28 (82.4)	
**Cesarean section**		6 (17.6)	3 (17.6)	3 (17.6)	
** Premature delivery,*n*(%)**	34	5 (15.2)	1 (6.3)	4 (23.5)	0.3353^F^
** Birth weight (Kg), median (IQR)**	34	2.88 (2.5, 3.3)	3.25 (2.7, 3.5)	2.7 (2.5, 3.0)	0.0364^W^
** Birth weight <2.5 Kg,*n*(%)**	34	6 (17.6)	2 (11.8)	4 (23.5)	0.6562^F^
**Anthropometrics at birth,*z*-score, mean (SD)**					
**Weight-for-age**	34	− 1.05 (1.34)	− 0.57 (1.41)	− 1.53 (1.12)	0.0356^T^
** Meconium microbiome Shannon diversity, median (IQR)**	26	1.66 (1.27, 2.18)	1.89 (1.33, 2.179)	1.32 (1.01, 2.26)	0.1134^W^
** Exclusive breastfeeding (6 months),*n*(%)**	34	30 (88.2)	15 (88.2)	15 (88.2)	1^F^
** Breastfeeding duration (months), median (IQR)**	34	9 (6, 10)	10 (9, 15)	6 (6, 9)	< .0001^W^
** Trimethoprim-sulfamethoxazole use (6 months), *n*(%)**	34	19 (55.9)	3 (17.7)	16 (94.1)	< .0001^F^
** Antibiotic use (6 months),*n*(%)**	34	25 (73.5)	8 (47.1)	17 (100)	0.0009^F^

^W^Wilcoxon’s, ^F^Fisher’s, ^T^*t* test

*HUU*, HIV unexposed uninfected; *HEU*, HIV exposed uninfected; *IQR*, interquartile range; *N*, number of participants; *SD*, standard deviation

To analyze potential variations between the breast milk of mothers with and without HIV, supervised analysis with orthogonal partial least square discriminant analysis (OPLS-DA) and unsupervised analysis with principal component analysis (PCA), were performed. A clear separation between breast milk collected at 6 weeks and breast milk collected at 6 months was seen (not separated by HIV infection; Fig. S10A), which was also observed in the PCA analysis (Fig. S10B). The metabolomics data were also analyzed by time point to identify potential differences by HIV infection. OPLS-DA showed a separation between the breast milk of mothers with and without HIV (Fig. S11AB), however, that separation was not as strong in the PCA analysis and did not reach significance for either of the two time points (Fig. S11CD).

Two-way repeated measures analysis of variance (ANOVA) (within subject) was used to identify significant differences in metabolites present in the breast milk of mothers with and without at 6 weeks and 6 months postpartum. ANOVA identified 106 metabolites that significantly differed between the two groups and time points (FDR *P* < 0.05; Table S2). Among the 106 metabolites, 16 were associated with HIV infection, 88 were associated with time point (6 weeks vs. 6 months postpartum), and two were associated with both HIV infection and time point. Within the 18 metabolites that were significantly different between the breast milk of mothers with and without HIV, 16 were higher and two were lower in the breast milk of mothers with HIV in comparison to breast milk from mothers without HIV (Table S2). The antiretroviral therapy (ART) drugs lamivudine and nevirapine were significantly more abundant in the breast milk of mothers with HIV. Kynurenine, which has received increasing attention due to its connection to inflammation, the immune system, and neurological conditions [37] was also significantly more abundant in breast milk from mothers with HIV. Tiglyl carnitine (C5), an acylcarnitine suggested to be involved in lipid metabolism in the brain, was present in significantly lower concentrations in the breast milk of mothers without HIV.

**Fig. 5:**
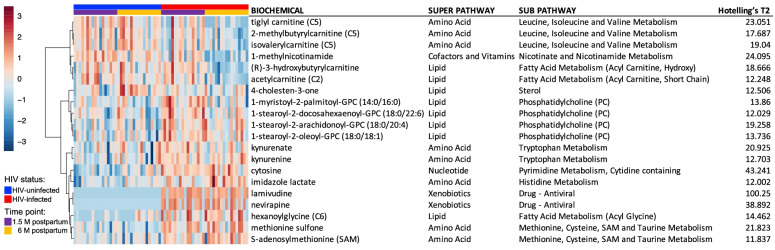
Heatmap of the 20 metabolites with the highest Hotelling’s *T*^2^ value. Heatmap of changes in breast milk metabolites at 6 weeks (purple) and 6 months (yellow) postpartum between mothers with (red) and without HIV (blue). The heatmap was created using the statistical package in MetaboAnalyst 5.0 (http://www.metaboanalyst.ca/MetaboAnalyst/). The heatmap is a visualization of the changes in abundance/level of breast milk metabolites (rows) for each mother (columns). The color ranges from dark red (high abundance or level) to dark blue (low abundance or level); white is no change

Multivariate empirical Bayes analysis (MEBA) was used to compare the time-course profiles between the breast milk of mothers with and without HIV. Metabolites with high Hotelling’s *T*^2^ values comprise those whose profiles are more different between the breast milk of mothers with and without HIV across the time points. The 20 metabolites with the highest Hotelling’s T^2^ value are represented in Fig. 5 as a heatmap. The time course profiles of the metabolites with the highest Hotelling’s *T*^2^ value are shown in Fig. S12. To determine whether the levels of any of these 20 metabolites are associated with the infants’ WAZ, Pearson’s correlations were run. After multiple comparison adjustment (FDR *P* value adjustment set at 0.05), none of the 20 metabolites exhibited a significant correlation with WAZ.

### Antiretroviral drugs found in breast milk of mothers with HIV are associated with a lower relative abundance of Bifidobacterium longum

Previous studies have shown that antiretrovirals administered to nursing mothers are present in their breast milk; however, the degree of antiretroviral transfer from mother to infant via breast milk and the downstream impact of infant antiretroviral drug exposure have not been well described [38–41]. Additionally, there is a lack of knowledge regarding the effect of antiretroviral drugs ingested via breast milk have on the infants' gut microbiota. To explore whether lamivudine and/or nevirapine in breast milk are associated with the relative abundance of any bacterial taxa in the infants’ gut microbiota, we performed logistic regressions. The results suggested that high nevirapine concentrations correlate with significantly lower relative abundances of *Bifidobacterium longum* in our cohort of 17 HEU infants (*P* = 0.040), consistent with our observation that breastfeeding infants born to mothers with HIV exhibit significantly less Bifidobacteria at 6 months postpartum than those born to mothers without HIV.

## Discussion

In sub-Saharan Africa, the scale-up of antiretroviral prophylaxis to prevent mother-to-child HIV transmission has dramatically reduced the number of children infected with HIV. Consequently, it is estimated that 15% of all infants born in sub-Saharan Africa are HEU [42]. Although not infected with HIV, this population remains at risk for early-life developmental abnormalities such as growth faltering [43, 44], increased morbidities [45–47], infant diarrhea [48, 49], and higher mortality during the first 12––24 months of life when compared to HUU infants [50–52].

Many of the mothers with HIV in this study opted not to breastfeed for long durations due to concerns for transmission of HIV to their babies (Fig. 2). Because breastfeeding is known to have a profound impact on the infant gut microbiota, our statistical analyses accounted for such differential feeding practices (newborn, breastfeeding, or not breastfeeding). The relative abundances of several bacterial taxa in the infant gut significantly differed based on breastfeeding status, one of which was *Bifidobacterium*, that was positively associated with breastfeeding. Early *Bifidobacterium* colonization of the infant gut is facilitated by the commencement of breastfeeding, a phenomenon that has been described in a number of other studies [24, 33, 53]. Members of the genus *Bifidobacterium* play an important role in the maturation of the infant gut by providing butyrate-producing colonic bacteria with exogenous acetate that can be used as a co-substrate to produce butyrate, a compound with anti-inflammatory properties that enhances intestinal barrier function and mucosal immunity [54–56]. Additionally, Bifidobacteria have been shown to metabolize tryptophan into indole-3-lactic acid and other beneficial metabolites in the infant gut [57].

Infants born to mothers with HIV exhibited lower WAZ from birth throughout the 18-month study period in comparison to HUU infants (Table 1, Fig. 4A). Our finding of lower WAZ at birth among HEU infants is consistent with several other studies conducted in African populations [27, 30, 58–60]. When our HEU infants were grouped by breastfeeding status, the breastfeeding HEU infants had significantly higher WAZ at 6 weeks postpartum compared to non-breastfeeding HEU infants (Fig. 4B). This result suggests that breastfeeding may be partially mitigating the adverse effects of maternal HIV status; however, this benefit did not persist as the difference in WAZ is no longer significant at 6 months postpartum when most of the mothers with HIV had stopped breastfeeding.

A few studies have suggested that fetal growth may be affected by in utero antiretroviral therapy (ART) drug exposure [28, 30, 60, 61] and that ART drugs are associated with preterm birth and low birth weight [62–65] and other adverse postnatal outcomes that may, in part, reflect the toxicity of nucleoside analogs used in ART [66, 67].

There are limited data reporting the concentrations of ART drugs in nursing infants as a result of transfer via breast milk [68, 69]; however, it has been shown that lamivudine and nevirapine [70, 71] are transferred to infants via breast milk in biologically significant concentrations [40]. Our breast milk metabolomics data showed that both lamivudine and nevirapine were present in the breast milk of our cohort of mothers with HIV and we observed that significantly lower levels of *Bifidobacterium longum* in the HEU infant gut microbiota correlated with high nevirapine concentration. However, there was no direct link between high ART drug concentrations and low WAZ, suggesting other factors could also be responsible for the adverse growth outcomes.

Besides the presence of ART drugs, we observed additional differences in the breast milk from mothers with and without HIV. Mothers with HIV had a significantly lower level of tiglyl carnitine (C5), an acylcarnitine, in their breast milk. Carnitine is involved in β-oxidation of fatty acids and plays other important roles in metabolism [72]. Carnitine deficiency has previously been reported to occur in adult patients with HIV [73, 74] and children [75], and may reflect gastrointestinal malabsorption. Acylcarnitine is present in relatively high levels in the brain [76] and can readily cross the blood–brain barrier [77]. Supplementation with acylcarnitine in neurological diseases [78] has been shown to be of benefit by enhancing lipid synthesis, altering and stabilizing membrane composition, modulating genes and proteins, improving mitochondrial function, increasing antioxidant activity, and enhancing cholinergic neurotransmission [79–82].

Another metabolite that was found at significantly higher levels in the breast milk of mothers with HIV was kynurenine. Kynurenine is a major metabolite of tryptophan (TRP), an essential amino acid that can only be acquired through diet in humans [83]. About 99% of TRP is metabolized via the kynurenine pathway (KP) [84], which contains several neuroactive metabolites that may influence brain function in health and disease [37], and it has been shown that the KP of TRP catabolism remains abnormally high in individuals with HIV [85, 86]. Because overexpression of this pathway has been associated with adaptive immune defects, it has been shown to have deleterious effects on disease progression and neurocognition in patients with HIV.

A major strength of this study is the longitudinal approach and large sample size. Two hundred seventy-two mother-infant-pairs were followed prenatally to 18 months postpartum (seven time points). Our results confirm data from earlier published reports that maternal HIV infection is associated with adverse growth outcomes of HEU infants [8, 9, 52, 58, 87–99]. Moreover, our data reveal that these differences persist from birth through at least 18 months, which is a critical window for the development and activation of the immune and central nervous systems [100]. Our results suggest that the interaction between maternal HIV status, the infant gut microbiota, breastfeeding, and growth outcome is complex. One of the most important observations in this study is that HEU infants exhibit growth deficits over the first 18 months of life when compared to HUU infants. While breastfeeding was shown to be of benefit to the HEU infants in this study, breastfed HEU infants still exhibited lower WAZ than the HUU infants. Unfortunately, the duration of breastfeeding by the HIV-positive mothers was relatively short, and we could not evaluate the potential impact of longer-term breastfeeding in this cohort. We also observed that the relative abundance of *Bifidobacterium* spp. was significantly lower in the gut microbiota of all HEU infants over the first 6 months postpartum, even if the HEU infants were receiving breast milk.

While our data provide a new level of understanding of the impact of maternal HIV infection and the potential role of the gut microbiota on infant health, additional follow-up studies are needed before any practical recommendations can be considered. Identification of the safest ART regimens for use in pregnancy that optimize both maternal and child outcomes still represents a key public health challenge. Breastfeeding was of benefit in our HEU infant cohort in the first weeks postpartum, however, ART drug metabolites in this cohort were associated with a lower abundance of *Bifidobacterium*, a genus that is essential in the development of a healthy infant gut microbiome and maturation of the immune system. We were not able to determine whether the association between ART drug metabolites and *Bifidobacterium* levels is a direct one, or an indirect one in which ART drug metabolites serve as markers of HIV infection. Therefore, a future study to evaluate the impact of ART drug metabolites in the breast milk of mothers with HIV may be indicated to better understand their effects on the maturation of the infant gut microbiota and/or immune activation markers. Given the lower relative abundance of *Bifidobacterium* in HEU infants, the use of a *Bifidobacterium* probiotic supplement may be of benefit in these populations [101]; however, this approach should be evaluated in a follow-up clinical trial targeting the critical window of the first 6 months postpartum.

One of the hypotheses at the start of this study was that we would uncover differences in the maternal microbiota related to HIV status. Therefore, it was somewhat unexpected that we did not observe significant differences in the vaginal and gut microbiota between mothers with and without HIV, although a similar finding was previously reported by Bender et al. [8]. As was proposed by Bender et al. [8], it may be that because of the HIV prophylaxis and/or prenatal care that was provided throughout the study, mothers may have been “too healthy” for any potential differences in the microbiota to be significant. The “healthfulness” of the mothers with HIV could have biased our results towards the null (i.e., no difference between mothers with and without HIV), which would mean the discovery of fewer differences that might exist in mothers with HIV that are not on ART therapy.

Nevertheless, there remains an urgent need to address the increased morbidity and mortality in HEU infants in the months following birth that have been described in numerous studies [8, 52, 58, 87–99]. While our data revealed a limited number of specific differences in the gut microbiota of HEU and HUU infants, they may be very important with respect to growth and development in the first 6 months of life. Another factor to consider in future studies is environmental enteropathy (or environmental enteric dysfunction), which affects predominantly children in low-income countries and is hypothesized to be caused by continuous exposure to fecal contamination in food, water, and fomites [102]. Thus, multiple additional efforts to strengthen the maternal and infant gut, including strategies to prevent or treat enteropathogen infections, should be a priority.

## Conclusions

Two hundred seventy-two mother-infant-pairs were followed prenatally to 18 months postpartum (seven time points). Our results confirm that maternal HIV infection is associated with adverse growth outcomes of HEU infants. Moreover, our data reveal that these differences persist from birth through at least 18 months, which is a critical window for the development and activation of the immune and central nervous systems. Our results suggest that the interaction between maternal HIV status, the infant gut microbiota, breastfeeding, and growth outcome is complex. We observed that the relative abundance of *Bifidobacterium* spp. was significantly lower in the gut microbiota of all HEU infants over the first 6 months postpartum, even if the HEU infants were receiving breast milk. Breastfeeding was of benefit in our HEU infant cohort in the first weeks postpartum, however, ART drug metabolites in this cohort were associated with a lower abundance of *Bifidobacterium*, a genus that is essential in the development of a healthy infant gut microbiome and maturation of the immune system. Evaluating the impact of ART drug metabolites in the breast milk of mothers with HIV is necessary to better understand their effects on the maturation of the infant gut microbiota and/or immune activation markers. The use of a *Bifidobacterium* probiotic supplement may be of benefit in these populations. Multiple additional efforts to strengthen the maternal and infant gut, including strategies to prevent or treat enteropathogen infections, should be a priority.

## Methods

### Study design, participant visit, and data collection

#### Design

This was a prospective cohort study of mother-infant pairs conducted at the University of Benin Teaching Hospital Nigeria (UBTH) between 2015 and 2018. The study was approved by the UBTH research ethics committee and the University of Maryland Baltimore Institutional Review Board.

#### Study participants

Pregnant women with and without HIV infection (~ 150 each) were recruited from the University of Benin Teaching Hospital located in Edo State, Southern Nigeria. Participating women were required to be aged between 18 and 45 years, have documented evidence of HIV status, and willing to comply with follow-up assessment schedule. Babies born to these women were also enrolled at birth. Recruited mother-infant pairs were assessed at birth and followed up for 18 months with scheduled assessment visits at 1, 6, 9, 15, and 18 months. Demographic, clinical, feeding, anthropometric and microbiome data were collected at each visit. Informed consent was also obtained from all mothers. University of Maryland Baltimore and UBTH Institutional Review Boards approved all study procedures.

#### Infant HIV testing

HIV DNA PCR test was done for all HEU babies at 6 weeks postpartum and at 4 months for non-breastfed infants or 2 months after breastfeeding cessation.

#### PMTCT antiretroviral regimen

About 70% of the mothers with HIV were already on highly active antiretroviral treatment (HAART) prior to the index pregnancy, and their triple regimens were continued. Others were initiated on antiretroviral drugs in line with Nigerian guidelines, which recommend HAART for women requiring treatment for their own disease or option B prophylaxis with triple regimen until 1 week after breastfeeding ceases, as well as nevirapine to the baby from birth to 6 weeks.

#### Clinical assessment

Standardized questionnaires were utilized at each study visit to document general medical and obstetric information, including medication and comorbidity history, general physical examination findings, and anthropometric assessment.

#### Feeding practice

Information on feeding practices was collected using structured feeding questionnaires. This included type, pattern, and duration of breastfeeding as well as complementary and alternate feeding practices.

#### Anthropometric assessment

Weight was measured to the nearest 0.1 Kg using “Salter Baby Scale (Model 180)” at birth and “Seca Digital Scale (Model 872)” subsequently. For the latter, baby’s weight was determined from the combined mother-baby weight measurement. Recumbent length was measured using an infantometer (“Seca 416”). A flexible non-elastic tape (“Seca 212”) was used to measure head and arm circumference. Low birth weight was defined as birth weight < 2.5 kg [103]. World Health Organization (WHO) child growth standards were used to generate z scores for weight for age (WAZ). WAZ ≤ 2 *z*-scores were defined as underweight [104, 105].

### Sample collection

#### Meconium and stool

About 0.5 g of meconium and stool samples were collected at birth and at each follow-up study visit respectively (Table S1). Similarly, 0.5 g of stool sample was collected from the mothers at enrollment and following delivery (Table S1).

#### Breast milk

Breast milk was collected by trained research nurses at 6 weeks and 6 months postpartum (Table S1). After washing hands with soap and water and cleaning the nipples and areolar area with cotton wool soaked in normal saline, 10 ml of breast milk was manually expressed and collected into a falcon tube. This was aliquoted into cryogenic vials and immediately stored at − 20 °C and later in − 80 °C freezers.

#### Vaginal swab

Vaginal swab was collected from the mothers at enrollment and again following delivery (Table S1). Specimen was collected using “Isohelix Sk-2” swab (Geneflow, Ltd, UK) following aseptic procedures. The swab was then inserted back into its container tube, the cap closed, and tube placed in a ziploc with ice pack, and this was subsequently stored at − 70 °C freezers (Table S1).

### DNA extraction and 16S rRNA gene sequencing

DNA was extracted from each fecal, meconium, and vaginal specimen. Both positive and negative controls (Zymo, Irvine, CA) were included in the DNA extraction process and the 16S rRNA gene sequence amplification process as previously described [106]. Samples were thawed at 4°C and, in aliquots of 0.15 g per tube, resuspended in 1 ml of 1 × phosphate-buffered saline. Cell lysis was initiated with two enzymatic incubations: 1. using 5 μl of lysozyme (10 mg/ml; Amresco, Solon, OH), 13 μl of mutanolysin (11.7 U/μl; Sigma-Aldrich), and 3 μl of lysostaphin (4.5 U/μl; Sigma-Aldrich) for an incubation of 30 min at 37 °C and, 2. using 10 μl of proteinase K (20 mg/ml; Research Products International, Mt. Prospect, IL), 50 μl of 10% SDS, and 2 μl of RNase (10 mg/ml) for an incubation of 45 min at 56 °C. After the enzyme treatments, cells were disrupted by bead beating in tubes with lysing matrix B (0.1-mm silica spheres; MP Biomedicals, Solon, OH), at 6 m/s at room temperature in a FastPrep-24 (MP Biomedicals). The resulting crude lysate was processed using the ZR fecal DNA miniprep kit (Zymo, Irvine, CA) according to the manufacturer’s recommendations. The samples were eluted with 100 μl of ultrapure water into separate tubes. DNA concentrations in the samples was determined with the Bioanalyzer 2100 DNA 1000 chip (Agilent, Santa Clara, CA).

### 16S rRNA gene sequence analysis

Hypervariable regions V3 and V4 of the bacterial 16S rRNA gene were amplified with primers 319F and 806R as previously described by [107, 108]*.* High-quality amplicon sequences were obtained on an Illumina HiSeq 2500 modified to generate 300 bp paired-end reads [108]. A total of 139 million reads were retained following chimera removal and 45,556 amplicon sequence variants (ASVs) were generated by DADA2 and taxonomically classified using the RDP Naïve Bayesian Classifier [109] trained with the SILVA v128 16S rRNA gene database [110]. ASVs of major stool taxa were assigned species-level taxonomy using speciateIT (http://ravel-lab.org/speciateit). Negative controls generated a negligible amount of sequencing reads, whereas the positive controls generated the expected mock community [106]. Taxa present at a relative abundance of less than 10^-5^ across all samples was removed from the dataset. The phyloseq R package [111] was used for analysis of the microbial community data.

### Sample preparation and ultra-high-performance liquid chromatography/mass spectrometry/mass spectrometry

A selection of 17 breast milk samples from mothers with HIV and 17 breast milk samples from mothers without HIV at visits 6 weeks and 6 months were shipped to Metabolon, Inc. (Durham, NC, USA) for metabolomics. The samples were chosen haphazardly, making sure the baseline characteristics of the mothers were similar and their infants were breastfed for at least 6 months postpartum (Table 2). Untargeted metabolite profiling was carried out by Metabolon Inc. (Durham, NC, USA) using ultra-high-performance liquid chromatography/mass spectrometry/mass spectrometry (UHPLC/MS/MS). Breast milk was mixed with methanol to recover chemically diverse metabolites after precipitating proteins. The methanol extract was divided into five fractions: two for analysis by two separate reverse-phase (RP) UPLC/MS/MS methods with positive ion mode electrospray ionization (ESI), one for analysis by RP/UPLC/MS/MS with negative ion mode ESI, one for analysis by hydrophilic interaction (HILIC) UPLC/MS/MS with negative ion mode ESI, and one sample was reserved for backup. The mass spectrometry (MS) analysis alternated between MS and data-dependent MS scans using dynamic exclusion. A pooled sample was created by taking a small aliquot from each of the samples, which served as technical replicates in the assay, whereas pure water samples served as a process blank, and a cocktail of quality control (QC) standards (Metabolon) was spiked into every standard sample to identify the instrument variability. The instrument variability determined by calculating the median relative standard deviation for the internal standards was 3%. The samples were randomized across the platforms, and internal standards and process blanks were added to each sample prior to injection into the mass spectrometers.

### Metabolomics data extraction, compound identification, and quantification

The raw data extraction, peak identification, and QC process were performed using Metabolon's proprietary hardware and software. The metabolites were identified using a proprietary in-house library based on standards that contained the retention time/index, mass to charge ratio, and chromatographic data (including MS/MS spectral data) on molecules present in the library. Additional mass spectral entries were created for structurally unnamed biochemicals, which were identified by their recurrent nature (both chromatographic and mass spectral). Peaks were quantified using the area under the curve. The biochemical data were normalized for the volume of breast milk used.

Raw data was extracted, peak-identified and QC processed using Metabolon’s hardware and software. Compounds were identified by comparison to library entries of purified standards or recurrent unknown entities. Metabolon maintains a library based on authenticated standards that contains the retention time/index, mass to charge ratio, and chromatographic data (including MS/MS spectral data) on all molecules present in the library. More than 3300 commercially available purified standard compounds have been acquired and registered into Metabolon’s system for analysis on all platforms for determination of their analytical characteristics. Additional mass spectral entries have been created for structurally unnamed biochemicals, which have been identified by virtue of their recurrent nature (both chromatographic and mass spectral). A data normalization step was performed to correct variation resulting from instrument inter-day tuning differences. Essentially, each compound was corrected in run-day blocks by registering the medians to equal one (1.00) and normalizing each data point proportionately (termed the “block correction”).

### Statistical analysis

Statistical analyses were performed using R (version 3.6.0). Demographic and clinical characteristics were compared between HEU and HUU children and their mothers using Wilcoxon, Fisher’s exact and t tests. Longitudinal comparisons of alpha diversity were performed using univariable and multivariable linear regression. Pairwise comparisons were performed with post hoc Tukey HSD test with FDR P value adjustment set at level 0.05. Principal coordinates analysis (PCoA) using Bray-Curtis dissimilarity was performed to assess the beta diversity. Permutational multivariate analysis of variance (PERMANOVA) was conducted to test whether the bacterial communities sequenced have different centroids based on HIV-status (mothers) or HIV-exposure (infants). Significance of the results was confirmed with a test of heterogeneity (ensure homogenous dispersion). In addition, multivariate association with linear models (MaAsLin2) [112], an additive general linear model with boosting that can capture the effects of a parameter of interest while deconfounding the effects of other metadata, was used to efficiently determine multivariable association between clinical metadata, 16S rRNA gene sequence data, and breast milk metabolomic data. MaAsLin2 analysis for the infants was adjusted for delivery type, prematurity, timepoint, antibiotic use, and breastfeeding at the time of visit. Additionally, MaAsLin2 parameters for taxa analysis were set as follows: *P* value control for Benjamini-Hochberg FDR was set at level 0.05, the minimum abundance for each taxon was set to 1% and the minimum percent of samples for which a taxon is detected at 1% was set to 10%. The parameters for the metabolomic analysis were as follows: *P* value control for Benjamini-Hochberg FDR was set at level 0.05, the minimum abundance for each metabolite was set to 0.001, and the minimum percent of samples for which a metabolite is detected at 0.001 was set to 10%. The heatmap was created using the statistical package in MetaboAnalyst 5.0 (http://www.metaboanalyst.ca/MetaboAnalyst/). ANOVA and post hoc test were performed by MetaboAnalyst 5.0. The *P* value was obtained by running the Fishers’ LSD after the ANOVA test and adjusted by multiple test corrections using the Benjamin-Hochberg procedure (FDR was set at level 0.05). Pearson's correlations were run using Benjamini-Hochberg multiple comparison adjustment (FDR *P* value adjustment set at 0.05).

## Supplementary Information

**Additional file 1: Figure S1**. Microbiota of 272 mother-infant pairs. **Figure S2**. Shannon diversity of 272 mother-infant pairs. **Figure S3**. PCoA of maternal microbiota based on HIV status. **Figure S4**. Shannon diversity of maternal microbiota based on HIV status. **Figure S5**. Maternal microbiota composition showed no significant differences based on HIV status. **Figure S6**. Number of breastfeeding and non-breastfeeding infants throughout the 18-month study period. **Figure S7**. PCoA of infant microbiomes based on HIV exposure, breastfeeding status, and time point. **Figure S8**. PCoA of infant microbiomes based on *Bifidobacterium longum* relative abundance, breastfeeding status, and time point. **Figure S9**. Shannon diversity of infant gut microbiota based on HIV exposure, breastfeeding status, and time point. **Figure S10**. Orthogonal partial least square discriminant analysis (OPLS_DA) and principal component analysis (PCA) of breast milk. **Figure S11**. Orthogonal partial least square discriminant analysis (OPLS_DA) and principal component analysis (PCA) of breast milk at six weeks postpartum and 6 months postpartum. **Figure S12**. Top 20 metabolites with highest Hotelling's T^2^ value. **Table S1**. Sample collection schedule. **Table S2**. Two-way repeated measures analysis of variance (ANOVA) of breast milk metabolites.

## Data Availability

Data and materials used in the analysis are available upon request from the corresponding authors for the purposes of reproducing or extending the analysis. Sequence reads from the 16S rRNA gene profiling are available at NCBI Sequence Read Archive under accession number PRJNA706727.

## Abbreviations

ART: Antiretroviral therapy
HEU: HIV-exposed uninfected
HUU: HIV-unexposed uninfected
IMC: Infant meconium
IST: Infant stool
IOS: Infant oral swab
MST: Maternal stool
MVS: Maternal vaginal swab
MBM: Maternal breast milk
PCoA: Principal coordinates analysis
PERMANOVA: Permutational multivariate analysis of variance
ASV: Amplicon sequence variant
ANOVA: Analysis of variance
FDR: False discovery rate
UPLC: Ultra-performance liquid chromatography
SI: Shannon index
MaAsLin2: Multivariable association with linear models
WAZ: Weight-for-age *z*-score
TMP-SMX: Trimethoprim-sulfamethoxazole
TRP: Tryptophan
KP: Kynurenine pathway
OPLS-DA: Orthogonal partial least square discriminant analysis
PCA: Principal component analysis
WHO: World Health Organization
MEBA: Multivariate empirical Bayes analysis
UHPLC/MS/MS: Ultra-high-performance liquid chromatography/mass spectrometry/mass spectrometry
RP: Reverse-phase
ESI: Electrospray ionization
MS: Mass spectrometry
QC: Quality control
HILIC: Hydrophilic interaction
