# Supplementary material for: Dynamics of the infant gut microbiota in the first 18 months of life: the impact of maternal HIV infection and breastfeeding
Source: Microbiome. 2022 Apr 12;10:61. doi: 10.1186/s40168-022-01230-1 (PMC9004197; doi:10.1186/s40168-022-01230-1)
Supplement: Supplementary file 2 — Additional file 1: Figure S1. Microbiota of 272 mother-infant pairs. Figure S2. Shannon diversity of 272 mother-infant pairs. Figure S3. PCoA of maternal microbiota based on HIV status. Figure S4. Shannon diversity of maternal microbiota based on HIV status. Figure S5. Maternal microbiota composition showed no significant differences based on HIV status. Figure S6. Number of breastfeeding and non-breastfeeding infants throughout the 18-month study period. Figure S7. PCoA of infant microbiomes based on HIV exposure, breastfeeding status, and time point. Figure S8. PCoA of infant microbiomes based on Bifidobacterium longum relative abundance, breastfeeding status, and time point. Figure S9. Shannon diversity of infant gut microbiota based on HIV exposure, breastfeeding status, and time point. Figure S10. Orthogonal partial least square discriminant analysis (OPLS_DA) and principal component analysis (PCA) of breast milk. Figure S11. Orthogonal partial least square discriminant analysis (OPLS_DA) and principal component analysis (PCA) of breast milk at six weeks postpartum and 6 months postpartum. Figure S12. Top 20 metabolites with highest Hotelling's T2 value. Table S1. Sample collection schedule. Table S2. Two-way repeated measures analysis of variance (ANOVA) of breast milk metabolites. [file 40168_2022_1230_MOESM2_ESM.docx]

**Supplementary Materials:**

**Fig. S1. Microbiota of 272 mother-infant pairs.**

**Fig. S1. Microbiota of 272 mother-infant pairs.** (A) PCoA plot of Bray-Curtis dissimilarity between maternal sample types (MST: maternal stool sample, MVS: maternal vaginal swab) revealed that each sample type clustered into distinct groups (PERMANOVA, *P* = 0.001). (B) PCoA plot of Bray-Curtis dissimilarity between infant sample types (IMC: infant meconium, IST: infant stool sample) revealed no significant difference between the two sample types.

**Fig. S2. Shannon diversity of 272 mother-infant pairs.**


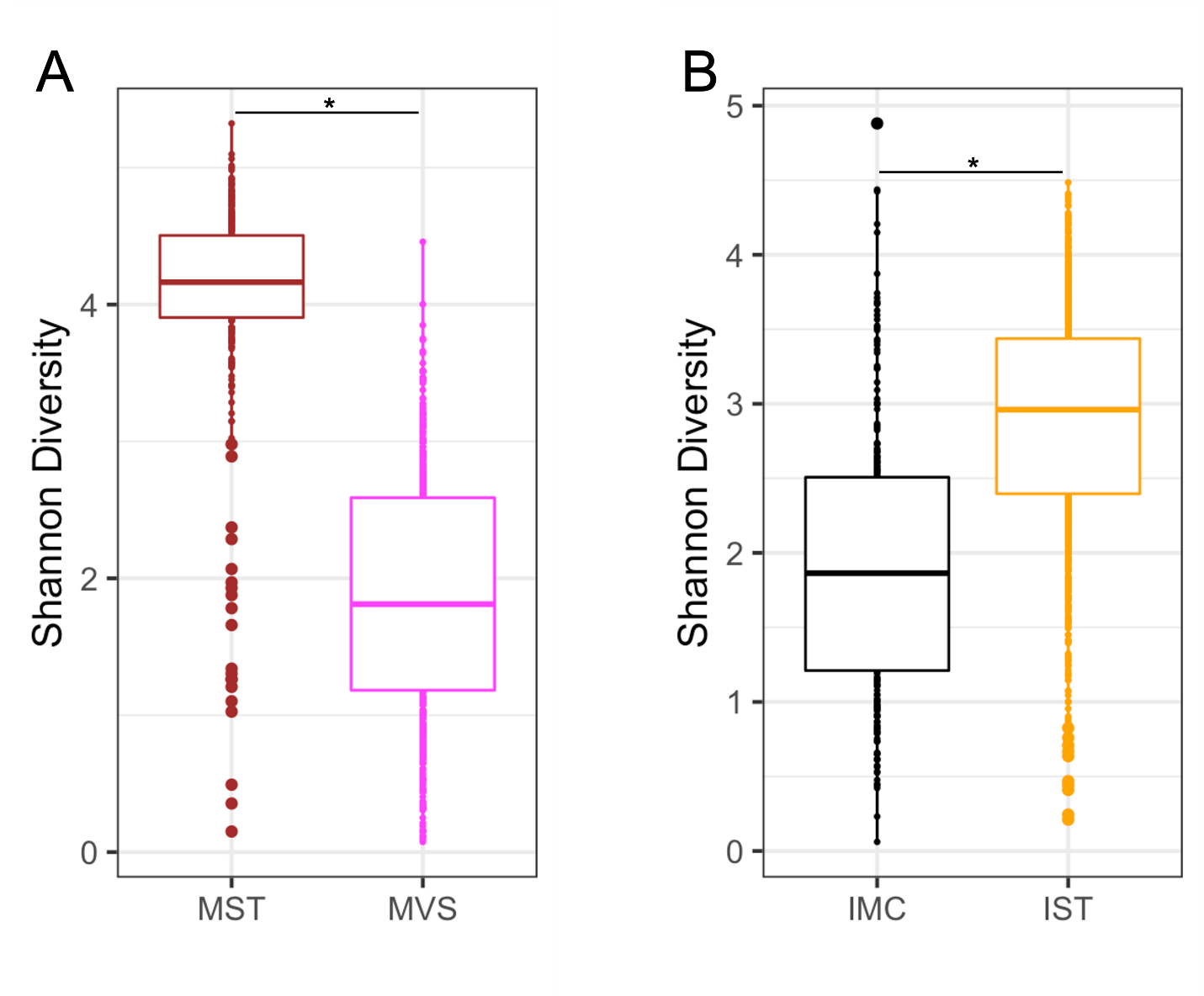


**Fig. S2. Shannon diversity of 272 mother-infant pairs.** (A) Alpha diversity was significantly different (Shannon index, *P* < 0.001) between mothers’ sample sites with MST exhibiting a higher diversity (Shannon index; SI = 4.06 ± 0.04 SEM) compared to MVS (SI = 1.85± 0.03 SEM). (B) Alpha diversity for the infants also displayed significant differences (Shannon index, *P* < 0.001); IMC had a lower diversity (SI = 1.92 ± 0.05 SEM) compared to IST (SI = 2.89 ± 0.02 SEM). Horizontal lines in boxplots indicate median, boxes show first and third quartiles.

**Fig. S3. PCoA of maternal microbiota based on HIV status.**

**Fig. S3. PCoA of maternal microbiota based on HIV status.** (A) PCoA plot of Bray-Curtis dissimilarity based on the mother's HIV status for stool samples revealed no significant separation. (B) PCoA plot of Bray-Curtis dissimilarity based on the mother's HIV status for vaginal swabs revealed no significant separation.

**Fig. S4. Shannon diversity of maternal microbiota based on HIV status.**

**Fig. S4. Shannon diversity of maternal microbiota based on HIV status. (**A) No significant differences in stool bacterial diversity were identified between HIV-infected and HIV-uninfected mothers. (B) No significant differences in vaginal bacterial diversity were identified between HIV-infected and HIV-uninfected mothers. Horizontal lines in boxplots indicate median, boxes show first and third quartiles.

**Fig. S5. Maternal microbiota composition showed no significant differences based on HIV status.**

**Fig. S5. Maternal microbiota composition showed no significant differences based on HIV status.** (A) Maternal stool (MST) bacterial composition for HIV-infected and HIV-uninfected mothers at the taxonomic level of genus (only genera made up with ASVs with a mean greater than 0.5% are shown). (B) Maternal vaginal (MVS) bacterial composition for HIV-infected and HIV-uninfected mothers at the taxonomic level of genus (only genera made up with ASVs with a mean greater than 0.5% are shown) prenatally and at birth. (C) Alpha diversity (Shannon index) for each sample type (MST: maternal stool, MVS: maternal vaginal swab) based on the mothers' HIV status (HIV-infected, HIV-uninfected) prenatally and at birth. Horizontal lines in boxplots indicate median, boxes show first and third quartiles.

**Fig. S6. Number of breastfeeding and non-breastfeeding infants throughout the 18-month study period.**

**Fig. S6. Number of breastfeeding and non-breastfeeding infants throughout the 18-month study period.** The median duration of breastfeeding in this study was 6 months; however, in HEU infants there was a significantly shorter duration of breastfeeding (median 1 month) in comparison to HUU infants (median 9 months) (*P* < 0.001).

**Fig. S7. PCoA of infant microbiomes based on HIV exposure, breastfeeding status, and time point.**

**
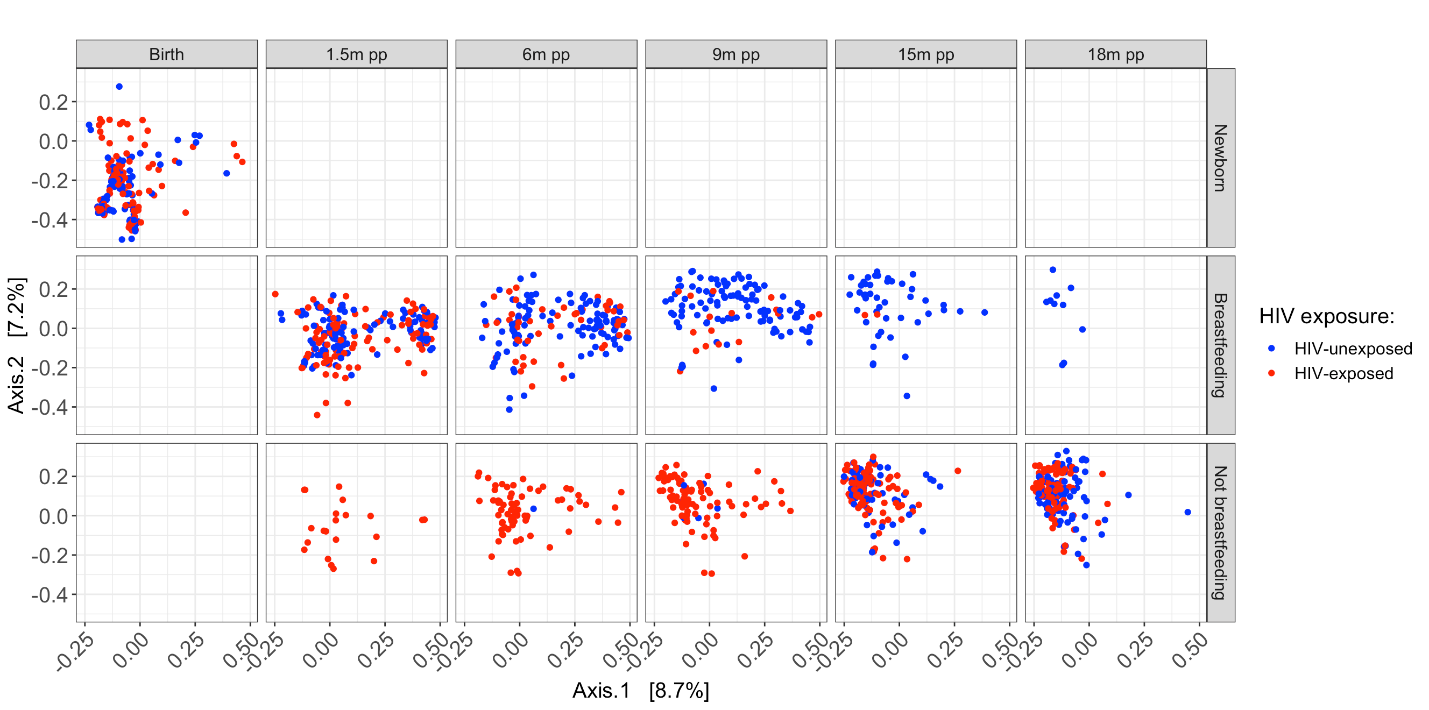
**

**Fig. S7. PCoA of infant microbiomes based on HIV exposure, breastfeeding status, and time point.** PCoA plot of Bray-Curtis dissimilarity based on HIV exposure, breastfeeding status, and time point revealed no significant separation between HEU and HUU infants' gut microbiota (PERMANOVA, all *P* > 0.05).

**Fig. S8. PCoA of infant microbiomes based on *Bifidobacterium longum* relative abundance, breastfeeding status, and time point.**

**
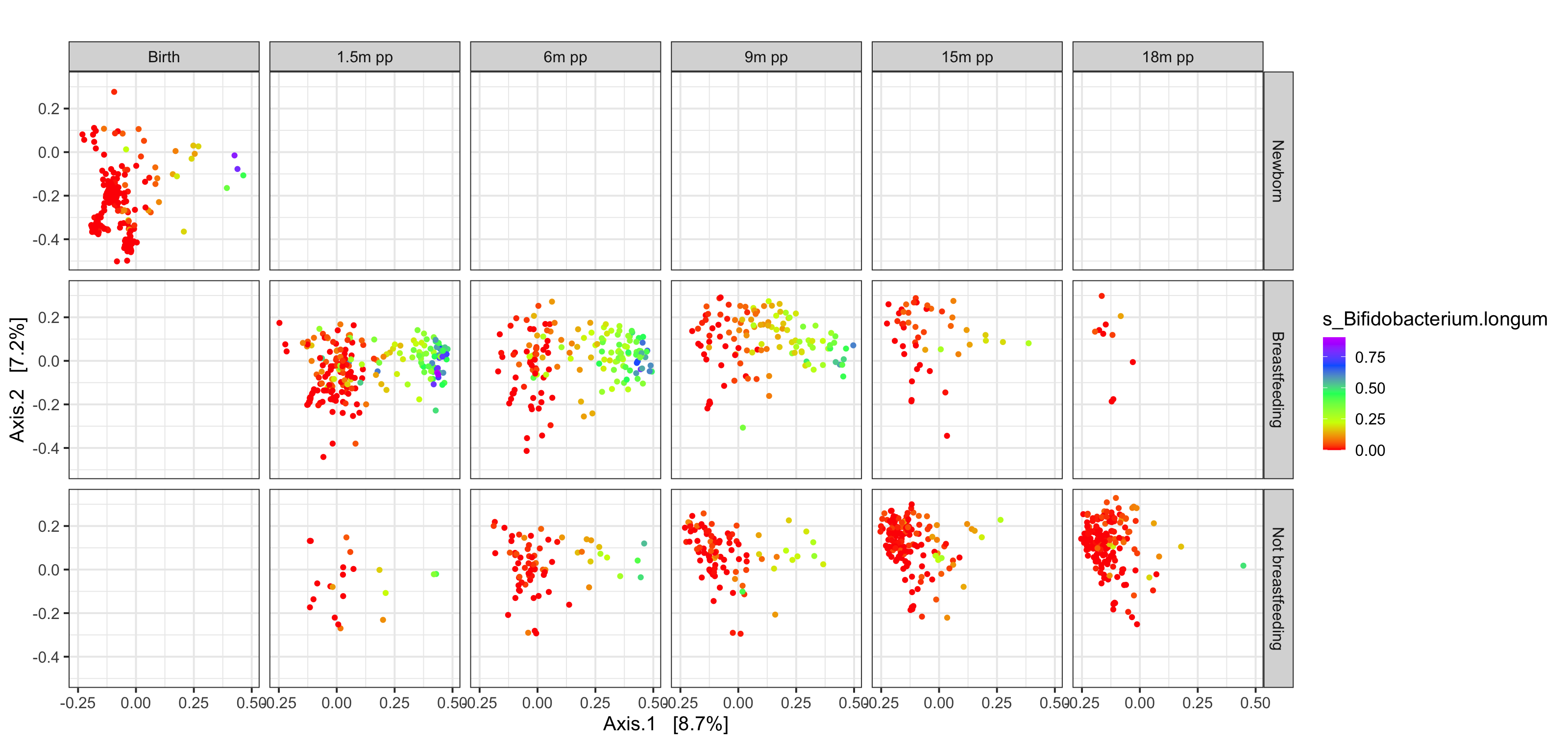
**

**Fig. S8. PCoA of infant microbiomes based on *Bifidobacterium longum* relative abundance, breastfeeding status, and time point.** PCoA plot of Bray-Curtis dissimilarity based on *Bifidobacterium longum* relative abundance, breastfeeding status, and time point revealed low *B. longum* containing gut microbiota samples to cluster to the left, whereas gut microbiota with high *B. longum* content gather to the right of the PCoA plot.

**Fig. S9. Shannon diversity of infant gut microbiota based on HIV exposure, breastfeeding status, and time point.**


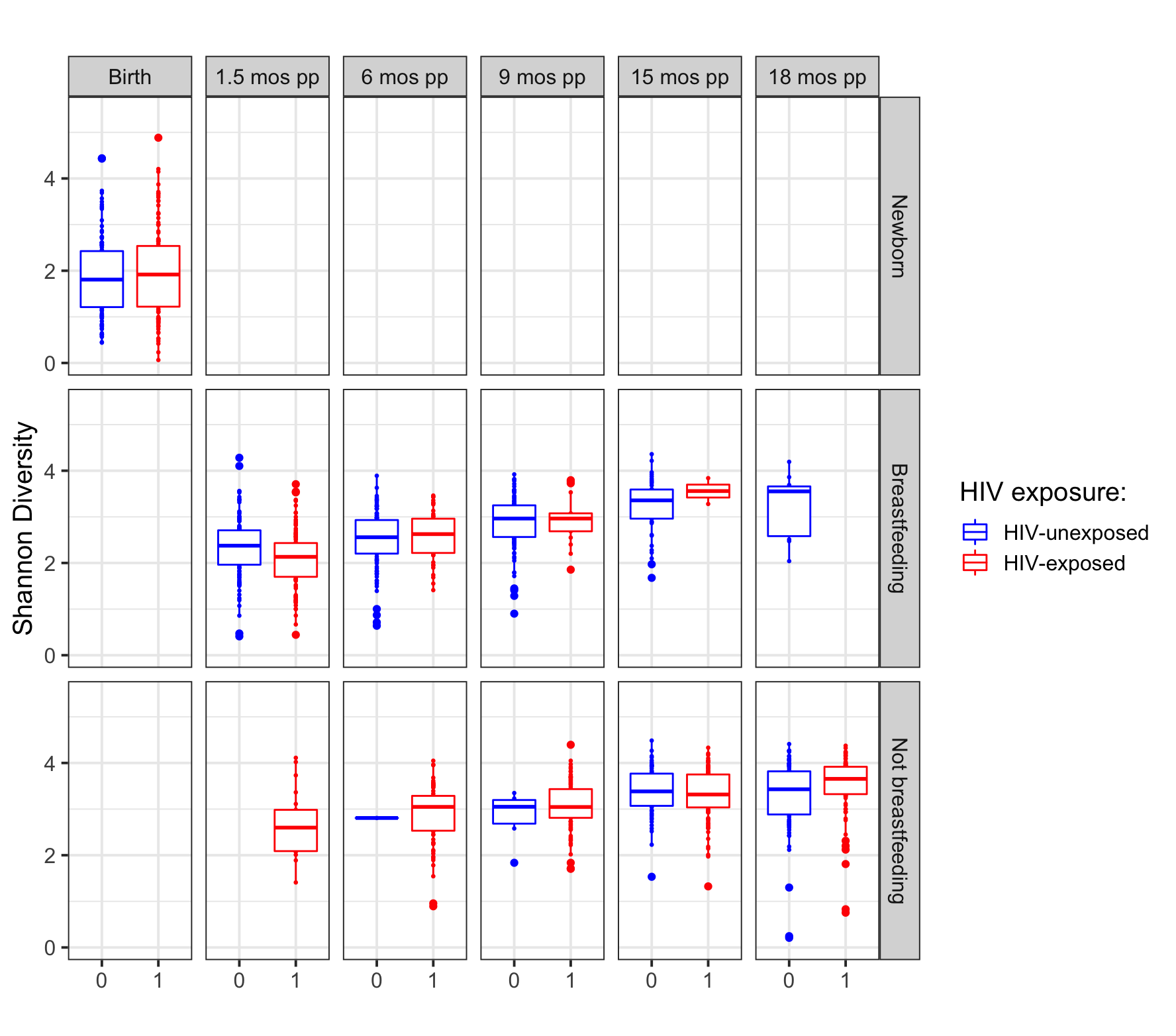


**Fig. S9. Shannon diversity of infant gut microbiota based on HIV exposure, breastfeeding status, and time point.** The bacterial diversity did not reach significance at any time point between HEU and HUU infants based on the breastfeeding status.

**Fig. S10. Orthogonal partial least square discriminant analysis (OPLS_DA) and principal component analysis (PCA) of breast milk.**

**Fig. S10. Orthogonal partial least square discriminant analysis (OPLS_DA) and principal component analysis (PCA) of breast milk.** (A) OPLS plot identified group clustering of breast milk samples collected at six weeks postpartum (blue) and six months postpartum (red). (B) PCA plot suggesting higher similarity between breast milk samples at six weeks postpartum (blue) when compared to breast milk samples collected at six months postpartum (red).

**Fig. S11. Orthogonal partial least square discriminant analysis (OPLS_DA) and principal component analysis (PCA) of breast milk at six weeks postpartum and six months postpartum.**

**Fig. S11. Orthogonal partial least square discriminant analysis (OPLS_DA) and principal component analysis (PCA) of breast milk at six weeks postpartum and six months postpartum.** (A) OPLS plot identified group clustering of breast milk samples collected at six weeks postpartum between HIV-infected (red) and HIV-uninfected (blue) mothers. (B) OPLS plot identified group clustering of breast milk samples collected at six months postpartum between HIV-infected (red) and HIV-uninfected (blue) mothers. (C) PCA plot suggesting slight differences between breast milk samples at six weeks postpartum between HIV-infected (red) and HIV-uninfected (blue) mothers. (D) PCA plot suggesting breast milk from HIV-infected (red) and HIV-uninfected (blue) mothers to be similar.

**Fig. S12. Top 20 metabolites with highest Hotelling's T^2^ value.**

**Fig. S12. Top 20 metabolites with highest Hotelling's T^2^ value.** Multivariate Empirical Bayes Analysis (MEBA) was used to compare the time-course profiles between HIV-infected and HIV-uninfected mothers' breast milk. Above are the time course profiles of the 20 metabolites with the highest Hotelling’s T^2^ value.

**Table S1. Sample collection schedule.**


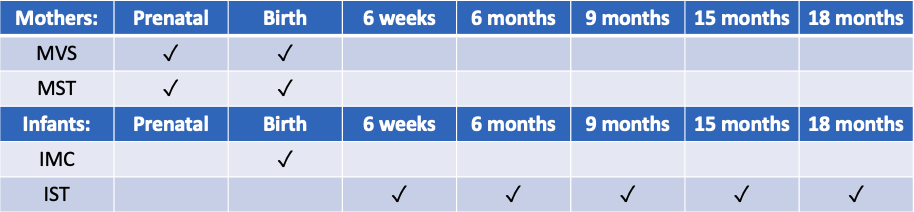


**Table S1: Sample collection schedule.** Samples were collected prenatally (MVS: maternal vaginal swab, MST: maternal stool swab), at birth (MVS, MST, IMC: infant meconium), six weeks postpartum (IST: infant stool swab), six months postpartum (IST), nine months postpartum (IST), 15 months postpartum (IST) and 18 months postpartum (IST).

**Table S2. Two-way repeated measures analysis of variance (ANOVA) of breast milk metabolites.**

**Table S2. Two-way repeated measures analysis of variance (ANOVA) of breast milk metabolites.** ANOVA identified 106 metabolites that significantly differed between the two groups and time points (FDR *P* < 0.05). Among the 106 metabolites, 16 were associated with HIV infection, 88 were associated with time point (six weeks vs. six months postpartum), and two were associated with both HIV infection and time point.
